# Development and evaluation of a duplex TaqMan qPCR assay for detection and quantification of *Trypanosoma cruzi* infection in domestic and sylvatic reservoir hosts

**DOI:** 10.1186/s13071-019-3817-9

**Published:** 2019-11-29

**Authors:** Diana P. Wehrendt, Andrea Gómez-Bravo, Juan C. Ramirez, Carolina Cura, Angélica Pech-May, Janine M. Ramsey, Marcelo Abril, Felipe Guhl, Alejandro G. Schijman

**Affiliations:** 1 Laboratorio de Biología Molecular de la Enfermedad de Chagas, INGEBI-CONICET, Buenos Aires, Argentina; 2 Fundación Mundo Sano, Buenos Aires, Argentina; 3 Centro Regional de Investigación en Salud Pública, Instituto Nacional de Salud Pública, Chiapas, México; 4 Universidad de los Andes, Bogotá, Colombia

**Keywords:** *Trypanosoma cruzi*, Chagas disease, Mammalian reservoirs, Molecular epidemiology, Internal amplification standard, Multiplex qPCR, Parasite load

## Abstract

**Background:**

A question of epidemiological relevance in Chagas disease studies is to understand *Trypanosoma cruzi* transmission cycles and trace the origins of (re)emerging cases in areas under vector or disease surveillance. Conventional parasitological methods lack sensitivity whereas molecular approaches can fill in this gap, provided that an adequate sample can be collected and processed and a nucleic acid amplification method can be developed and standardized. We developed a duplex qPCR assay for accurate detection and quantification of *T. cruzi* satellite DNA (satDNA) sequence in samples from domestic and sylvatic mammalian reservoirs. The method incorporates amplification of the gene encoding for the interphotoreceptor retinoid-binding protein (IRBP), highly conserved among mammalian species, as endogenous internal amplification control (eIAC), allowing distinction of false negative PCR findings due to inadequate sample conditions, DNA degradation and/or PCR interfering substances.

**Results:**

The novel TaqMan probe and corresponding primers employed in this study improved the analytical sensitivity of the assay to 0.01 par.eq/ml, greater than that attained by previous assays for Tc I and Tc IV strains. The assay was tested in 152 specimens, 35 from 15 different wild reservoir species and 117 from 7 domestic reservoir species, captured in endemic regions of Argentina, Colombia and Mexico and thus potentially infected with different parasite discrete typing units. The eIACs amplified in all samples from domestic reservoirs from Argentina and Mexico, such as *Canis familiaris*, *Felis catus*, *Sus scrofa*, *Ovis aries*, *Equus caballus*, *Bos taurus* and *Capra hircus* with quantification cycles (Cq’s) between 23 and 25. Additionally, the eIACs amplified from samples obtained from wild mammals, such as small rodents *Akodon toba*, *Galea leucoblephara*, *Rattus rattus*, the opossums *Didelphis virginiana*, *D. marsupialis* and *Marmosa murina*, the bats *Tadarida brasiliensis*, *Promops nasutus* and *Desmodus rotundus*, as well as in *Conepatus chinga*, *Lagostomus maximus*, *Leopardus geoffroyi*, *Lepus europaeus*, *Mazama gouazoubira* and *Lycalopex gymnocercus*, rendering Cq’s between 24 and 33.

**Conclusions:**

This duplex qPCR assay provides an accurate laboratory tool for screening and quantification of *T. cruzi* infection in a vast repertoire of domestic and wild mammalian reservoir species, contributing to improve molecular epidemiology studies of *T. cruzi* transmission cycles.

## Background

Chagas disease, a neglected tropical disease caused by the protozoan parasite *Trypanosoma cruzi* is endemic in Latin America, where it is mainly transmitted by hematophagous insects belonging to the genera *Triatoma*, *Rhodnius*, *Pastrongylus* and *Mepraia.* Other transmission routes, such as congenital (from mother to child), oral (by consuming contaminated food) and through blood transfusions and organ transplantation, are also important. Approximately seven million people are estimated to suffer from Chagas disease and hundreds of thousands of infected individuals have migrated to non-endemic countries [1].

The natural cycles of transmission involve sylvatic, domestic and peridomestic habitats. Opossums, armadillos and rodents are major sylvatic reservoir hosts, whereas humans, dogs, cats and commensal (synanthropic) rodents are the main hosts in domestic or peridomestic habitats [2–4]. A major question of epidemiological relevance is whether these types of transmission cycles are connected or independent. Characterizing the level of interconnection/ independence of these transmission cycles is paramount to trace the origins of (re)emerging cases in areas under vector or disease surveillance [5, 6].

Assessing the infection status of potential mammalian reservoirs is essential. Molecular techniques, such as qPCR have much greater sensitivity than conventional parasitological methods [7–11]. However, the diverse composition of biological samples collected from different mammalian reservoir species may affect amplification accuracy, making it difficult to compare prevalence of infection among different species within a same area under study and/or between different geographical regions. Blood samples may contain substances acting as qPCR inhibitors, leading to false negative results and sub-estimated prevalence rates. The quality of the sample may be altered during transportation from the site of blood collection to the molecular biology laboratory and/or during DNA purification. Accordingly, an accurate method must include an internal amplification control. In this context, we aimed to develop a duplex qPCR assay which would allow for simultaneous amplification of a *T. cruzi* DNA specific target and an endogenous internal control (eIAC) as amplification standard. The design of novel TaqMan probe and primers targeting a satellite DNA (satDNA) sequence allowed for improved analytical sensitivity, beyond that of other previously developed assays based on the same target [12, 13], especially for TcI and TcIV strains [14]. The eIAC was based on a gene fragment encoding the interphotoreceptor retinoid-binding protein (IRBP), which is highly conserved among mammalian species and its usefulness as a DNA integrity control was previously reported in a conventional PCR assay [15]. Once standardized, the duplex assay has been evaluated in a panel of biological samples collected from different sylvatic and domestic mammalian species captured in field studies at endemic areas of Argentina, Colombia and Mexico.

## Methods

### Mammalian reservoir samples

#### Analysis of a standard panel of samples

A first evaluation of the duplex *T. cruzi* satDNA/IRBP qPCR assay (index test) was carried out using archival DNA from blood samples of well-characterized domestic and sylvatic mammalian species previously tested using standardized qPCR procedures (comparator test, [13]) in order to estimate their agreement.

#### Analysis of field samples

The index method was assayed using DNA extracted from peripheral blood samples preserved in guanidine hydrochloride 6M, EDTA 0.2 M (GE), pH 8.00 (blood:GE proportion of 1:3) and collected from domestic and sylvatic mammalian reservoirs captured in endemic regions from Argentina, Colombia and Mexico.

Argentinean wild and domestic samples were provided by Andrea Gomez-Bravo (Fundación Mundo Sano, Buenos Aires, Argentina) from Añatuya, Santiago del Estero, Argentina. Colombian samples were provided by Felipe Guhl (Universidad de los Andes, Bogotá, Colombia). Mexican samples were collected from mammalian reservoirs captured in an endemic region for Chagas disease in Yucatán, Mexico and kindly provided by Janine M. Ramsey (Centro Regional de Investigación en Salud Pública, Chiapas, Mexico).

### DNA extraction methods

DNA was extracted from 300 µl of whole blood/GE samples (blood:GE proportion of 1:3) using phenol-chloroform based purification (for Mexican samples) or the High Pure PCR Template Preparation Kit (Roche Diagnostic Corp., Indiana, USA), following manufacturer instructions (for Argentinean and Colombian samples).

### Design of an endogenous internal amplification standard for duplex qPCR

**Fig. 1:**
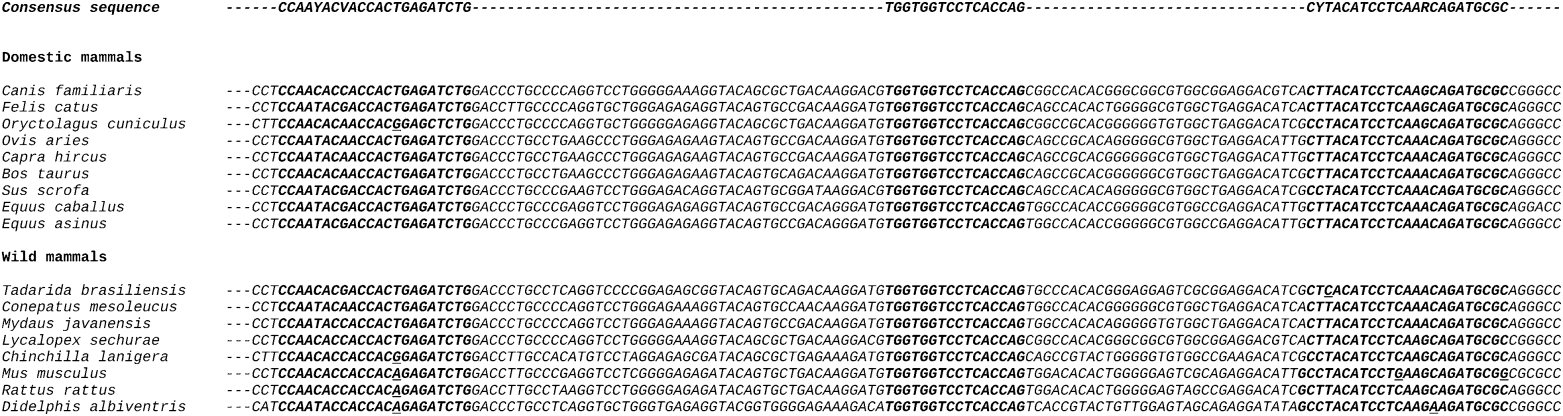
IRBP sequence alignment for different wild and domestic reservoir species. Primer and probe annealing sequences are highlighted in bold letters. Nucleotides that differ from the primer or probe sequences are underlined

A pair of primers and a TaqMan probe complementary to a highly conserved region within the amplified zone of the highly conserved mammalian IRBP gene were designed. Primer IRBP2 Fw was modified with respect to primer IRBP-CF-FWD reported by Ferreira et al. [15] for molecular diagnosis of leishmaniasis. Primer IRBP3Rv and probe IRBPTq were designed from a consensus sequence obtained after the alignment of IRBP sequences, available from 9 domestic and 8 wild mammalian reservoir species on GenBank (Table 1, Fig. 1).

**Table 1:** Primer and probe sequences and concentrations used in duplex TaqMan qPCR assay for detection of *T. cruzi* DNA in mammalian reservoir species

Gene target	Primer/probe name	Primer/probe sequence (5’-3’)	Reaction concentration (µM)	Amplicon size (bp)	Source
Satellite repeat unit from *T. cruzi* genome	Primer Cruzi 1c	TGAATGGYGGGAGTCAGAG	0.75	98	Ramírez et al. [14]
	Primer Cruzi 2c	ATTCCTCCAAGMAGCGGAT	0.75		
	Probe Cruzi 3	FAM-CACACACTGGACACCAA-NFQ-MGB	0.05		
IRBP gene from mammalian genome	Primer IRBP2 Fw	CCAAYACVACCACTGAGATCTG	0.60	140	Present study
	Primer IRBP3 Rv	GCGCATCTGYTTGAGGATGTARG	0.60		
	Probe IRBP Tq	HEX-TGGTGGTCCTCACCAG-NFQ-MGB	0.05

### Duplex TaqMan qPCR assay

The reaction was performed in a final volume of 20 µl with FastStart Universal Probe Master Mix (Roche Diagnostics, Mannheim, Germany) and 5 µl of DNA, in a Rotor-Gene 3000 (Corbett Life Science, Cambridge, UK) or an ABI 7500 (Applied Biosystems, Foster City, CA) device. For *T. cruzi* DNA amplification, new primers Cruzi1c, Cruzi2c [14] and probe Cruzi3 were used to enhance sensitivity with respect to a previous satDNA qPCR [12], in particular for Tc I and Tc IV strains. An internal amplification standard was amplified using primers IRBP Fw and Rv and IRBP probe. Their sequences and final concentrations in the qPCR reaction are given in Table 1. Cycling conditions were an initial step of 10 min at 95 °C and 45 cycles at 95 °C for 15 s and 56 °C for 1 min.

### Analytical parameters of duplex *T. cruzi* satDNA/IRBP qPCR assay

The satDNA single qPCR reaction was inclusive for strains belonging to discrete typing units DTUs TcI to TcVI, as previously reported [14]. To assess analytical sensitivity of the duplex format, blood from non-infected dogs was spiked with cultured epimastigotes of CL Brener and Silvio X10 *T. cruzi* strains (TcVI and TcI, respectively) to a final concentration of 10^7^ parasite equivalents/ml (par.eq/ml) and treated with three volumes of guanidine hidrochloride 6 M-EDTA 0.2 M (pH 8.00) (GE). Next, 10-fold serial dilutions were performed to cover a range between 0.001 to 10^6^ par.eq/ml. DNA of each dilution was purified and amplified in duplicate by duplex qPCR. Theoretical *versus* measured Cq values were converted to log10 par.eq/ml and plotted for linear regression analysis. Analytical sensitivity was estimated using triplicate dilutions of the above-mentioned spiked samples for both parasites and analytical specificity was estimated using DNA from *T. rangeli*, *Leishmania major*, *L. donovani* and *L. amazonensis.*

### Quality controls of the duplex *T. cruzi* satDNA/IRBP qPCR assay

Each DNA extraction round included one blood sample from a seronegative dog as a negative extraction control. Each amplification round included two positive DNA controls containing 1 par.eq/ml and 100 par.eq/ml of CL Brener spiked dog samples and one non-template control.

The satDNA/IRBP qPCR results were considered as valid when the Cq of IRBP was within the expected range according to the criteria of Tukey: Cq’s.75th percentile + 1.5 × interquartile distance of median Cq, which would indicate inhibition or material loss in samples from the same experiment with *n *> 10 [16].

### *Trypanosoma cruzi* DNA quantification of satDNA/IRBP qPCR positive samples

A panel of 22 satDNA/IRBP qPCR-positive samples was quantified for estimation of parasite load. For this, a standard quantification curve was constructed. Given that satDNA qPCR positive samples were genotyped as TcI [17, 18], DNA was obtained from non-infected dog blood spiked with 10^7^ par.eq/ml of Silvio X10 clone (TcI) cultured parasites, and serially diluted in DNA obtained from blood collected from non-infected dogs aiming to cover a range of standards containing 10^−1^ to 10^5^ par.eq/ml.

### Data analysis

To compare the agreement between the index duplex qPCR assay with comparator standardized qPCR procedures in a panel of characterized samples, inter-observer kappa coefficients were calculated using GraphPad Software online statistical calculators (http://www.graphpad.com/quickcalcs/kappa1.cfm). Kappa values < 0.01 indicate no agreement, those between 0.1 and 0.4 indicate weak agreement, those between 0.41 and 0.60 indicate clear agreement, those between 0.61 and 0.80 indicate strong agreement, and those between 0.81 and 1.00 indicate nearly perfect agreement.

## Results

### Design and analytical performance of duplex *T.cruzi* satDNA/IRBP qPCR assay

IRBP primer and probe sequences were designed from a consensus IRBP sequence obtained after alignment of orthologous sequences from different mammalian species, available in the GenBank database (Table 1, Fig. 1). The reportable range of *T. cruzi* satDNA/IRBP qPCR assay was assessed in single and duplex formats (Additional file 1: Figure S1). No significant differences between single *T. cruzi* satDNA qPCR and duplex *T. cruzi* satDNA /IRBP qPCR were observed when comparing the Cq values obtained for different *T. cruzi* DNA concentrations ranging between 10 and 10^5^ par.eq/ml (Additional file 1: Figure S1).

The duplex *T. cruzi* sat DNA/IRBP qPCR analytical sensitivity was evaluated in dog blood samples spiked with cultured parasites from the Silvio X10 (TcI) and CL Brener (TcVI) stocks. The reportable range was from 0.1 to 10^5^ par.eq./ml and from 1 to 10^4^ par.eq./ml for CL Brener and Silvio X10 stocks, respectively. Analytical sensitivities were 0.01 par.eq/ml for both *T. cruzi* stocks.

The assay amplified exclusively *T. cruzi* DNA samples; in contrast, it did not amplify DNA from different *Leishmania* species and *T. rangeli* (Table 2). Moreover, we compared the agreement between the duplex *T. cruzi* sat DNA/IRBP qPCR assay with previously reported PCR procedures in a panel of well-characterized blood samples from domestic and sylvatic mammal reservoirs (Table 3). An almost perfect agreement (% of agreement: 97.83%; Cohen’s k: 0.95) was obtained.

**Table 2:** Analytical parameters of the duplex *T.cruzi* satDNA/IRBP qPCR assay

Analytical parameter	satDNA/IRBP qPCR
Analytical sensitivity, par.eq./ml	
TcI (Silvio X10)	0.01
TcVI (CL Brener)	0.01
Inclusivity (fg/µl)	
Tc I (Silvio X10)	0.25
Tc II (Y)	0.125
Tc III (M5631 cl5)	0.0625
Tc IV (4167)	0.0625
Tc V (MnCl2)	0.0625
Tc VI (CL Brener)	0.0625
Exclusivity (non detectable qPCR) pg/µl	
*T. rangeli*	100
*L. major*	1000
*L. donovani*	1000
*L. amazonensis*	100
Reportable range	
Cl Brener	0.1–10^5^ par.eq/ml; y = − 2.48X + 22.69; *R*^2^ = 0.99
Silvio X10	1–10^4^ par.eq/ml; y = − 2.64 + 27.28; *R*^2^ = 0.98

**Table 3:** Comparison of *T. cruzi* DNA detection by means of *T. cruzi* satDNA/IRBP qPCR assay (index test) and standardized qPCR (comparator test)

Panel of well-characterized samples	*n*	Index test	
		Positive	Negative
Comparator qPCR test-positive			
*Canis familiaris*	2	2	0
*Felis catus*	5	5	0
*Ovis aries*	3	3	0
*Didelphis* sp.	6	6	0
Total	16	16	0
Comparator qPCR test-negative			
*Canis familiaris*	9	0	9
*Felis catus*	1	0	1
*Ovis aries*	3	1	2
*Capra hircus*	9	0	9
*Promops nasutus*	6	0	6
*Conepatus chinga*	1	0	1
*Lagostomus maximus*	1	0	1
Total	30	1	29

*Note*: Comparator qPCR test was reported in Ramirez et al. [13]

### Evaluation of blood samples from wild and domestic reservoirs

Blood sample panels from different mammalian species captured in three endemic regions for Chagas disease (Santiago del Estero, Argentina; Maní, Colombia; and Yucatán, México) were tested for simultaneous detection of *T. cruzi* infection and IRBP amplification (Table 4).

**Table 4:** IRBP (eIAC) gene amplification in duplex satDNA/IRBP qPCR assay for *T.cruzi* DNA detection in samples from reservoir species

	*n*	satDNA/IRBP qPCR			
		IRBP control			*T cruzi* positive/total
		Valid samples	Mean Cq	SD	
Wild reservoirs (*n* = 35)					
Small rodents					
*Akodon toba*, Argentina	5	5	26.29	2.22	0/5
*Galea leucoblephara*, Argentina	1	1	26.88	nd	0/1
*Rattus rattus*, Argentina	1	1	24.72	nd	0/1
Marsupials					
*Didelphis virginiana*, México	4	4	24.29	1.27	0/4
*Didelphis marsupialis*, Colombia	3	3	26.28	0.27	3/3
*Marmosa murina*, Colombia	3	3	29.73	4.91	3/3
Bats					
*Tadarida brasiliensis*, Argentina	1	1	24.48	nd	0/1
*Promops nasutus*, Argentina	4	4	33.60	1.97	0/4
*Desmodus rotundus* (vampire), Argentina	2^b^	1	27.90	nd	0/1
Other mammals					
*Conepatus chinga* (skunk), Argentina	3	3	24.86	1.65	0/3
*Lagostomus maximus* (viscacha), Argentina	3	3	28.38	0.20	0/3
*Leopardus geoffroyi* (wildcat), Argentina	1	1	22.71	nd	0/1
*Lepus europaeus* (hare), Argentina	1	1	27.88	nd	0/1
*Mazama gouazoubira* (brown brocket deer), Argentina	1	1	26.78	nd	0/1
*Lycalopex gymnocercus* (Pampas fox), Argentina	2	2	25.08	0.60	0/2
Domestic reservoirs (*n* =117)					
*Bos taurus* (cow), Argentina	12	12	24.84	0.31	0/12
*Canis lupus familiaris* (dog), Argentina	27	27	24.52	1.10	0/27
*Canis lupus familiaris* (dog), México^a^	4	4	28.85	4.50	4/4
*Capra hircus* (goat), Argentina	24	24	23.75	1.13	0/24
*Equus caballus* (horse), Argentina	2	2	23.40	0.49	0/2
*Felis catus* (cat), Argentina	4	4	26.21	0.37	0/4
*Felis catus* (cat), México	10	10	26.45	0.61	10/10
*Ovis aries* (sheep), Argentina	29	29	25.16	0.92	0/29
*Ovis aries* (sheep), México^a^	2	2	32.67	1.51	2/2
*Sus scrofa domesticus* (pig), Argentina	3	3	22.72	0.98	0/3

^a^ Phenol chloroform DNA extraction

^b^ One sample was IRBP negative

*Note*: IRBP amplification and detection of *T.cruzi* positive cases are shown

*Abbreviations*: SD, standard deviation; Cq, quantification cycle; nd, not determined

The eIAC amplified in all samples from domestic reservoirs from Argentina and Mexico, such as *Canis familiaris*, *Felis catus*, *Sus scrofa*, *Ovis aries*, *Equus caballus*, *Bos taurus* and *Capra hircus* with Cq’s between 23 and 25. It also amplified samples from wild mammals from Argentina, Colombia and Mexico, such as small rodents *Akodon toba*, *Galea leucoblephara*, *Rattus rattus*, the opossums *Didelphis virginiana*, *D. marsupialis* and *Marmosa murina*, the bats*, Tadarida brasiliensis*, *Promops nasutus* and *Desmodus rotundus*, as well as in *Conepatus chinga* (skunk), *Lagostomus maximus* (viscacha), *Leopardus geoffroyi* (wildcat), *Lepus europaeus* (hare), *Mazama gouazoubira* (brown brocket deer) and *Lycalopex gymnocercus* (Pampas fox) rendering Cq’s between 24 and 33 (Table 4). The Tukey’s criterion [16] was used to detect samples with outlier Cq values for the eIAC, which would indicate PCR inhibition or material loss in samples from the same experiment with *n* > 10. Only one sample of *Desmodus rotundus* was considered invalid.

Samples from Colombian *Didelphis marsupials*, *Marmosa murina*, Mexican dogs, cats and sheep that showed amplification of *T. cruzi* satDNA were considered positive (Table 4, [14]).

### Quantification of parasite load

**Fig. 2:**
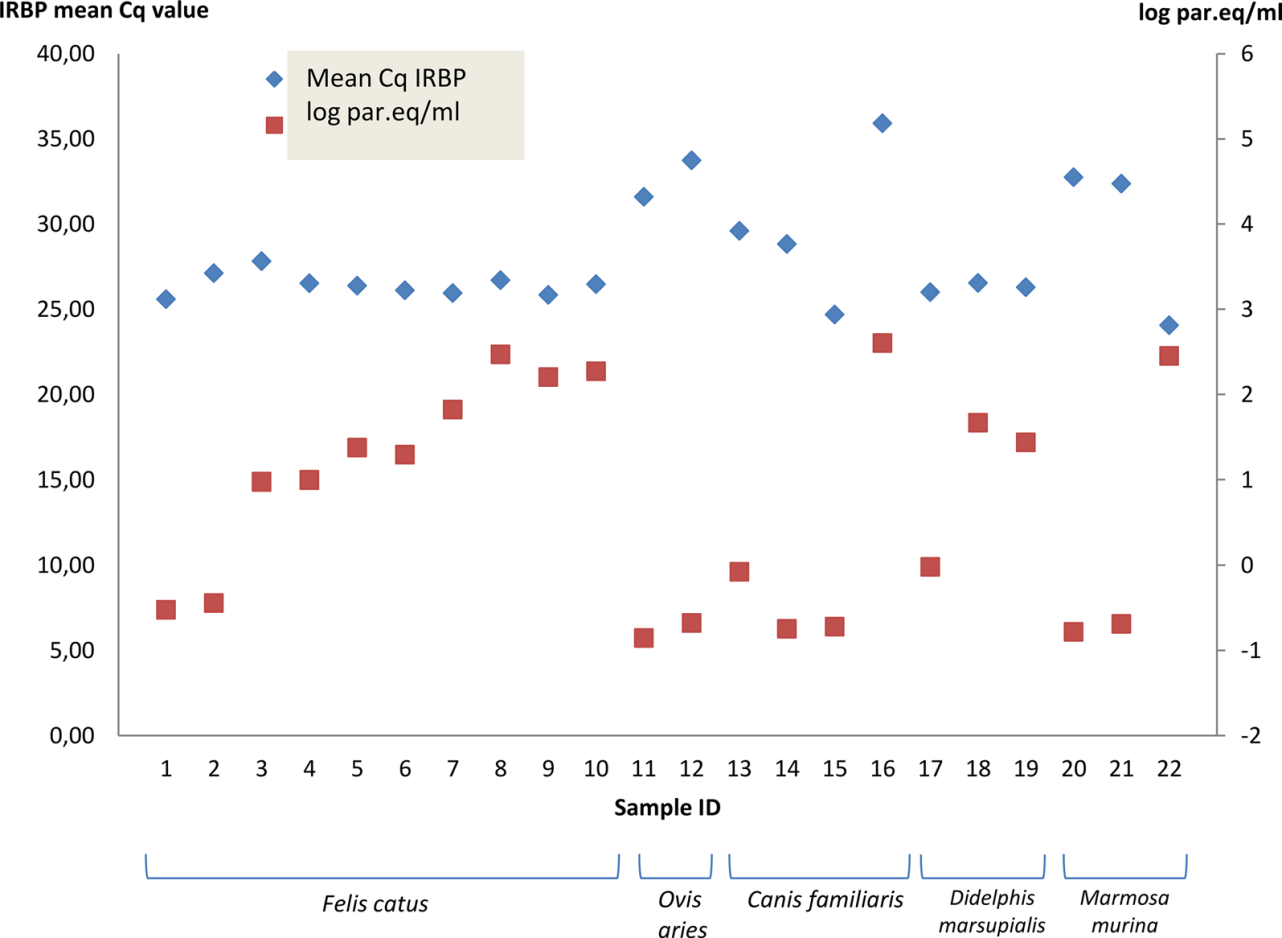
Quantification of parasite loads by means of duplex *T. cruzi* satDNA/IRBP qPCR in infected specimens. Quantification is expressed in parasite equivalents/ml of blood

TaqMan qPCR allows for quantification of parasitic burden in infected samples. Parasite loads were quantitated in infected sheep, dog and cat samples, as well as in *Didelphis marsupialis* and *Marmosa murina* (*n* = 22) (Fig. 2, Table 5). Except for both *Ovis aries* specimens, individuals from the other species presented heterogeneity in their parasite loads, ranging from 0.14 to 4.02 10^2^ par.eq/ml.

**Table 5:** Parasite loads in satDNA/IRBP qPCR-positive samples from mammalian reservoir hosts

Sample ID	Mammalian species	Duplex SatDNA/IRBP qPCR				DTU
		Mean Cq IRBP	Mean Cq cruzi	par.eq/ml	log par.eq/ml	
1	*Felis catus*	25.59	28.70	0.30	− 0.522878745	TcI
2	*Felis catus*	27.13	28.52	0.36	− 0.443697499	TcI
3	*Felis catus*	27.82	24.78	9.49	0.977266212	TcI
4	*Felis catus*	26.53	24.73	9.94	0.997386384	TcI
5	*Felis catus*	26.39	23.75	23.72	1.375114685	TcI
6	*Felis catus*	26.11	23.96	19.67	1.29380436	TcI
7	*Felis catus*	25.96	25.58	66.40	1.822168079	TcI
8	*Felis catus*	26.70	20.89	296.45	2.471951455	TcI
9	*Felis catus*	25.84	21.58	160.17	2.204581176	TcI
10	*Felis catus*	26.48	21.40	187.50	2.273001272	TcI
11	*Ovis aries*^a^	31.61	29.45	0.14	− 0.853871964	TcI/TcVI
12	*Ovis aries*^a^	33.74	29.13	0.21	− 0.677780705	TcI/TcVI
13	*Canis familiaris*^a^	29.60	27.47	0.84	− 0.075720714	TcVI
14	*Canis familiaris*^a^	28.83	29.31	0.18	− 0.744727495	TcI/TcVI
15	*Canis familiaris*^a^	24.69	29.27	0.19	− 0.721246399	TcI/TcVI
16	*Canis familiaris*	35.92	18.23	402.73	2.60500859	TcI
17	*Didelphis marsupialis*	26.01	26.80	0.96	− 0.017728767	nd
18	*Didelphis marsupialis*	26.54	21.29	46.41	1.666564777	nd
19	*Didelphis marsupialis*	26.30	22.04	27.39	1.437592032	nd
20	*Marmosa murina*	32.76	29.28	0.17	− 0.782516056	nd
21	*Marmosa murina*	32.37	29.17	0.21	− 0.688246139	nd
22	*Marmosa murina*	24.07	18.72	284.38	2.453891414	nd

^a^ DNA extracted with phenol-chloroform

*Abbreviation*: nd, not determined

## Discussion

*Trypanosoma cruzi* transmission cycles in sylvatic and domestic mammals have been studied in different eco-epidemiological settings in endemic regions [5]. Initially, microscopic analysis, blood culture or xenodiagnosis were used for detection and isolation of *T. cruzi* strains from mammalian reservoirs [19, 20]. Later, studies developed in-house conventional amplification procedures for direct detection and genotyping of *T. cruzi* in domestic and wildlife reservoirs [6–9, 11, 21, 22] whereas in dogs qPCR assays were carried out [23, 24]. However, methods lacking internal amplification controls could not discriminate between absence of infection and inadequate samples. Here, we have developed a TaqMan-based duplex qPCR procedure useful for detection and quantification of parasite loads in biological samples from wild and domestic mammalian reservoirs, coupled with an IRBP-DNA-based internal amplification standard. This enables a distinction between true negative samples and false negative samples due to the presence of PCR interfering substances and/or degradation of DNA.

The assay was evaluated using 35 blood samples from 15 different wild reservoir species and 117 samples from 7 domestic mammalian species. The IRBP-DNA-based integrity control performed adequately in all above mentioned specimens except in one DNA sample from *Desmodus rotundus.* The IRBP Cq-values were variable in different species, in particular in wild reservoirs, which may arise from different concentrations of nucleated cells in the blood of the different species [25, 26] and/or a different yield in blood-based DNA. In the case of column-based DNA extraction procedures, the pigs and wildcats presented the lowest IRBP Cq-values (22.72 and 22.71, respectively) whereas the bat (*Promops nasutus*) showed the highest Cq-values (32.86). Nevertheless, in some cases, samples of the same species extracted using different DNA purification methods yielded different Cq-values for IRBP-DNA. In particular, phenol-chloroform-extracted DNA samples from Mexico yielded higher between-sample mean Cq- and SD-values than those obtained using column-based DNA extraction (Table 4). Previous comparison of DNA extraction methods from blood samples showed that those based in organic solvents rendered a higher degree of PCR inhibition [27, 28]. Thus, in comparative studies of parasitic burden among individuals from the same species and/or among other reservoir species, the same DNA extraction procedure should be used and the acceptable range of IRBP Cq-values should be estimated for each round and method of DNA extraction in order to detect outlier Cq-values that allow distinction of false negative samples [16]. We have explored the capacity of the IRBP internal control to detect DNA degradation by performing the following experiments: incubation of DNA samples for 48 hours at room temperature and exposition of DNA samples to UV light. In both cases the Cq-values of IRBP increased compared to outlier values (unpublished results).

On the other hand, high parasite loads may inhibit amplification of IRBP. This will not be a problem for qualitative detection of *T. cruzi* infection, but if accurate parasite load quantifications are required, it is recommended to dilute the clinical sample and repeat the qPCR assay to achieve IRBP Cq-values within the acceptable range.

The high analytical sensitivity for satDNA amplification for both CL Brener and Silvio X10 stocks obtained with our assay had not been previously achieved, especially for TcI and TcIV strains [12, 13]. Probably, the exhaustive alignment for satDNA sequences performed in this study, including a higher number of strains for each *T. cruzi* stock, contributed in this regard [14]. Indeed, to our knowledge, this is the first time that this set of satDNA-based PCR primers and probe have been used in biological specimens.

## Conclusions

Our results indicate this novel assay is useful for *T. cruzi* infection screening in samples from different mammalian species, either in prospective studies or employing archival DNA. Sample quality can be inferred by means of eIAC amplification. Moreover, the quantification of parasite load may be indicative of the severity and the stage of infection in these reservoir species and their “transmission potential” in their habitats, thus contributing to epidemiological knowledge of factors involved in *T. cruzi* transmission cycles.

## Supplementary information

**Additional file 1: Figure S1.** Comparison of single and duplex *T. cruzi* satDNA qPCR reportable ranges for detection and quantification of *T. cruzi* DNA.

## Data Availability

Data supporting the conclusions of this article are included within the article and its additional file. All raw data are available upon request to the corresponding authors or at http://ingebi-conicet.gov.ar/biologia-molecular-de-la-enfermedad-de-chagas/.

## Abbreviations

qPCR: real time polymerase chain reaction
satDNA: satellite DNA
IRBP: interphotoreceptor retinoid-binding protein
eIAC: endogenous internal amplification control
Cq: quantification cycle
par.eq/ml: parasite equivalents per milliliter
UV light: ultraviolet light
SD: standard deviation
DTU: discrete typing unit
GE: guanidine hydrochloride/EDTA
