# Supplementary material for: Development and evaluation of a duplex TaqMan qPCR assay for detection and quantification of Trypanosoma cruzi infection in domestic and sylvatic reservoir hosts
Source: Parasit Vectors. 2019 Nov 29;12:567. doi: 10.1186/s13071-019-3817-9 (PMC6884757; doi:10.1186/s13071-019-3817-9)
Supplement: Supplementary file 1 — Additional file 1: Figure S1. Comparison of single and duplex T. cruzi satDNA qPCR reportable ranges for detection and quantification of T. cruzi DNA. [file 13071_2019_3817_MOESM1_ESM.docx]

**Additional file 1: Figure S1**
